# Fish feeds supplemented with calcium-based buffering minerals decrease stomach acidity, increase the blood alkaline tide and cost more to digest

**DOI:** 10.1038/s41598-022-22496-3

**Published:** 2022-11-02

**Authors:** Harriet R. Goodrich, Alex A. Berry, Daniel W. Montgomery, William G. Davison, Rod W. Wilson

**Affiliations:** 1 Institute for Marine and Antarctic Studies, University of Tasmania, Private Bag 49, Hobart, TAS 7001 Australia; 2 Biosciences, University of Exeter, Exeter, Devon, EX4 4PS UK; 3 Department of Zoology, 4200 - 6270 University Blvd, Vancouver, BC Canada

**Keywords:** Zoology, Ecology, Metabolism, Respiration, Conservation biology, Ecophysiology, Ecosystem ecology, Freshwater ecology

## Abstract

Predatory fish in the wild consume whole prey including hard skeletal parts like shell and bone. Shell and bone are made up of the buffering minerals calcium carbonate (CaCO_3_) and calcium phosphate (Ca_3_(PO_4_)_2_). These minerals resist changes in pH, meaning they could have physiological consequences for gastric acidity, digestion and metabolism in fish. Using isocaloric diets supplemented with either CaCO_3_, Ca_3_(PO_4_)_2_ or CaCl_2_ as non-buffering control, we investigated the impacts of dietary buffering on the energetic cost of digestion (i.e. specific dynamic action or SDA), gastric pH, the postprandial blood alkalosis (the “alkaline tide”) and growth in juvenile rainbow trout (*Oncorhynchus mykiss*). Increases in dietary buffering were significantly associated with increased stomach chyme pH, postprandial blood HCO_3_^−^, net base excretion, the total SDA and peak SDA but did not influence growth efficiency in a 21 day trial. This result shows that aspects of a meal that have no nutritional value can influence the physiological and energetic costs associated with digestion in fish, but that a reduction in the SDA will not always lead to improvements in growth efficiency. We discuss the broader implications of these findings for the gastrointestinal physiology of fishes, trade-offs in prey choice in the wild, anthropogenic warming and feed formulation in aquaculture.

## Introduction

Digestion and assimilation of ingested food incur an energetic cost to the animal known as the specific dynamic action (SDA). The SDA arises as a result of the physical, biochemical and physiological processes necessary to capture, breakdown and assimilate a meal^1–4^. Meal type, size, feeding frequency, and environmental conditions like temperature, salinity, and hypoxia are all known to affect the magnitude, duration and peak of the SDA^3,5–9^. How these factors influence the SDA, relates to how they affect the physiological, biochemical or mechanical costs associated with digestion. For example, feeds with a higher protein content elicit a greater SDA due to the costs associated with protein synthesis^10^, while liquid, cooked or soft tissue meals result in a reduced SDA due to the lesser requirement for mechanical breakdown in the stomach^7,11^. Recently, dietary buffering (ability to resist changes in acidity) and diet acidity have been shown to have a significant effect on the SDA in juvenile barramundi (*Lates calcarifer*) due to impacts on gastric acid secretion and the recovery of acid–base homeostasis following feeding^12^.

During feeding and digestion gastric hydrochloric acid (HCl) secretion is necessary for the activation of proteolytic enzymes in the stomach which break down long chain amino acids^13^. Acid-secretion by gastric oxyntopeptic cells (the equivalent of parietal cells in mammals) is driven by the intracellular reversible hydration-dehydration reaction of CO_2_:

$${\text{CO}}_{2} + {\text{H}}_{2} {\text{O}} \rightleftharpoons {\text{HCO}}_{3}^{ - } + {\text{H}}^{ + }$$

Acid secretion consumes energy directly through the use of H^+^/ K^+^-ATPase in the oxyntopeptic cells that line the stomach in most fish, with one ATP consumed by H^+^/K^+^ ATPase for every H^+^ pumped across into the stomach lumen^14–16^. As a result, for every O_2_ consumed, gastric H^+^/K^+^ ATPase will pump a maximum of 5 H^+^ into the gastric lumen, but due to the inevitable back-leak of protons, the efficiency is likely to be lower (e.g. 2.3 H^+^ per O_2_ consumed^16^). This same reaction will also create equimolar amounts of bicarbonate (HCO_3_^−^) within oxyntopeptic cells^17^. In order to maintain intracellular acid–base balance, excess cellular HCO_3_^−^ is transferred into the blood across the basolateral membrane. The entry of HCO_3_^−^ into the blood causes a rapid rise in blood pH and HCO_3_^−^ concentration following feeding, a phenomenon known as the post-prandial alkaline tide^18–20^. Freshwater fish are able to balance this blood alkalosis by excreting most of the excess HCO_3_^−^ via the gills^18.^ This consumes further energy because the net excretion of HCO_3_^-^ into the water involves the basolateral extrusion of H^+^ into the blood by vacuolar H^+^-ATPase^21^.

Aspects of a meal that buffer the acidity of the stomach contents should therefore influence gastric acid secretion, the alkaline tide and recovery of acid–base balance following feeding^12^. For wild fish with true acidic stomachs consuming whole prey, significant buffering components would come from the calcium minerals in bone and shell. Rainbow trout (*Oncorhynchus mykiss*) are an important commercial fin fish that are known to feed on a variety of vertebrate and invertebrate prey types in nature^22,23^. In the wild, rainbow trout prey on bony fishes, crustaceans, insects and gastropods^23^. When calcium carbonate from shell (CaCO_3_) and calcium phosphate from bone (Ca_3_(PO_4_)_2_) dissolve within the stomach, their extra buffering will require additional gastric acid secretion which could enhance the physiological and energetic costs associated with digestion in fish.

This concept presents a significant knowledge gap, considering that the maintenance of an acidic stomach is an energy-consuming process, and diets containing minerals that resist changes in acidity may require more acid and, in turn, energy to digest. Not only could this influence the magnitude and duration of the SDA response, but it has previously been suggested that an increase in the energy required for digestion could have impacts on fish growth^4,24^. To address this knowledge gap, the present study aimed to measure the SDA, alkaline tide, base excretion and growth of juvenile (100—200 g) rainbow trout fed on isocaloric pelleted diets that differed only in the calcium salt added (CaCO_3_, Ca_3_(PO_4_)_2_, or CaCl_2_ [as a non-buffering control with the same calcium content]). It was hypothesised that the digestion of a highly buffered feed will lead to increased acid secretion in the stomach, a more pronounced alkaline tide and a greater net bicarbonate excretion to the surrounding water. It was predicted that these increased energy demands would produce a greater SDA and result in reduced fish growth efficiency.

## Results

### Gut pH and intestinal HCO_3_^–^ concentration after feeding

**Figure 1:**
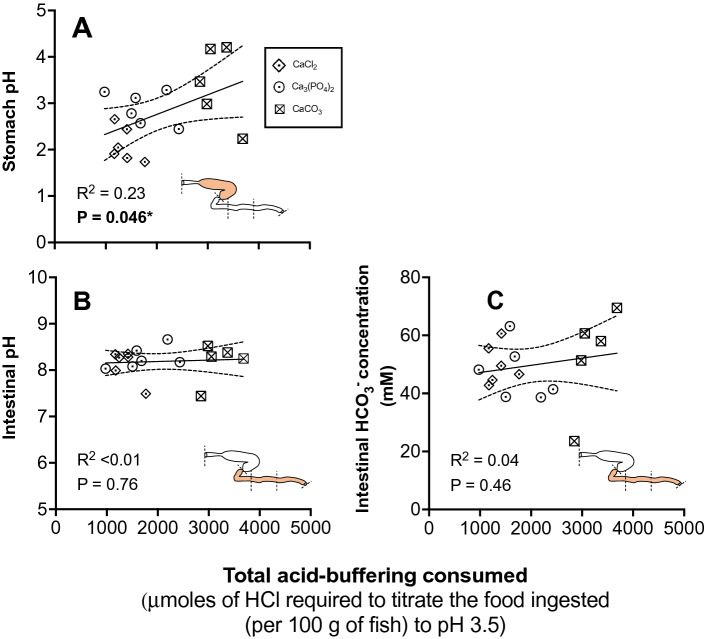
Change in stomach pH (**A**), intestinal pH (**B**) and intestinal HCO_3_^−^ concentration (**C**) of juvenile rainbow trout (*Oncorhynchus mykiss*) 48 h after consuming a 2.5% ration of one of three experimental feeds (n = 6 for the CaCl_2_ and Ca_3_(PO_4_) treatments; *n* = 5 for CaCO_3_ treatment) of varying buffer capacity. The ranked order (and relative magnitude) of the dietary acid-buffering capacity of each diet was CaCO_3_ > Ca_3_(PO_4_)_2_ > CaCl_2_ (2.4 > 1.4 > 1). Solid line represents the regression line while the dotted line represents the 95% confidence interval (CI). Significance was accepted at *P* < 0.05 following a simple linear regression. Each data point represents values from individuals.

At 48 h after feeding stomach pH significantly increased with dietary buffer capacity (R^2^ = 0.23, *P* = 0.046) (Fig. 1A). Stomach pH increased from pH 2.10 ± 0.15 in fish consuming the CaCl_2_ diet treatment to pH 3.41 ± 0.37 in fish consuming the CaCO_3_ diet treatment. Tukey’s multiple comparisons reveal that stomach pH significantly differed between fish consuming the CaCO_3_ and CaCl_2_ diet treatment only (F_2, 14_ = 8.32, *P* < 0.01).

All fish showed high intestinal pH (> 8) and concentrations of HCO_3_^−^ (~ 50 mM) that was similar across diet treatments (pH: R^2^ < 0.01, *P* = 0.76, HCO_3_^–^: R^2^ = 0.04, *P* = 0.46; (pH: F_2, 14_ = 0.26, *P* = 0.78, HCO_3_^–^: F_2, 14_ = 0.30, *P* = 0.75) (Fig. 1 B,C). The average intestinal pH was 8.12 ± 0.13, 8.26 ± 0.09 and 8.17 ± 0.19 in the CaCl_2_, Ca_3_(PO_4_)_2_ and CaCO_3_ feeding treatments respectively, while intestinal HCO_3_^–^ was 50.0 ± 2.8, 47.2 ± 3.9 and 52.6 ± 7.8 mM in the CaCl_2_, Ca_3_(PO_4_)_2_ and CaCO_3_ feeding treatments, respectively.

### The alkaline tide

**Figure 2:**
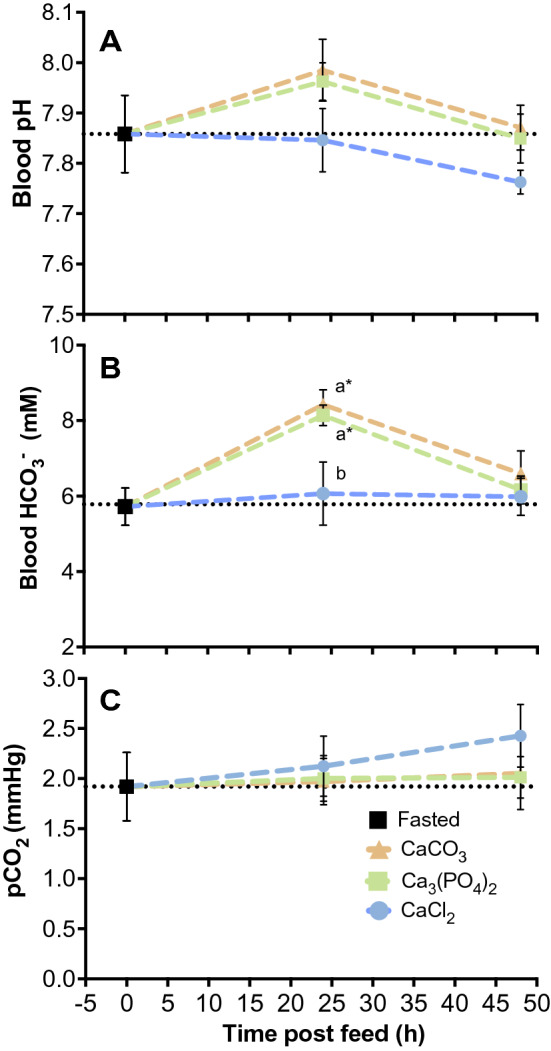
The average change in blood pH (A), plasma HCO_3_^−^ concentration (B) and pCO_2_ (C) over time in juvenile rainbow trout (n = 40) (*Oncorhynchus mykiss*) fed voluntarily on a 2.5% ration of one of three diets supplemented with either CaCl_2_, CaCO_3_ or Ca_3_(PO_4_)_2_. Data are expressed as means ± SE. Different letters (abc) indicate significant differences between groups at 24 or 48 h post feed following a one-way ANOVA and Tukey multiple comparisons test. Significance was accepted at *P* < 0.05.

At 24 h post feed fish fed the CaCO_3_ and Ca_3_(PO_4_)_2_ diet treatments experienced an increase in blood HCO_3_^−^ concentration of 32 and 29%, or 2.7 and 2.4 mM, respectively. However, the corresponding rise in blood pH of ~ 0.15–0.17 units was not significant in either treatment (Fig. 2A, B), and by 48 h these blood acid–base variables were back to pre-feeding levels (see Table S1). Fish feeding on the control CaCl_2_ diet did not experience any change in blood HCO_3_^−^ concentration or pH following feeding and values from this group remained similar to pre-feeding levels for the entire measurement period (*P* > 0.05—see Supplementary Table S1). Blood pCO_2_ was unaffected by feeding and remained similar across all diet treatments and time points post feed (Fig. 2C) (*P* > 0.05 see Supplementary Table S1 and S2).

### Net acid–base fluxes

**Figure 3:**
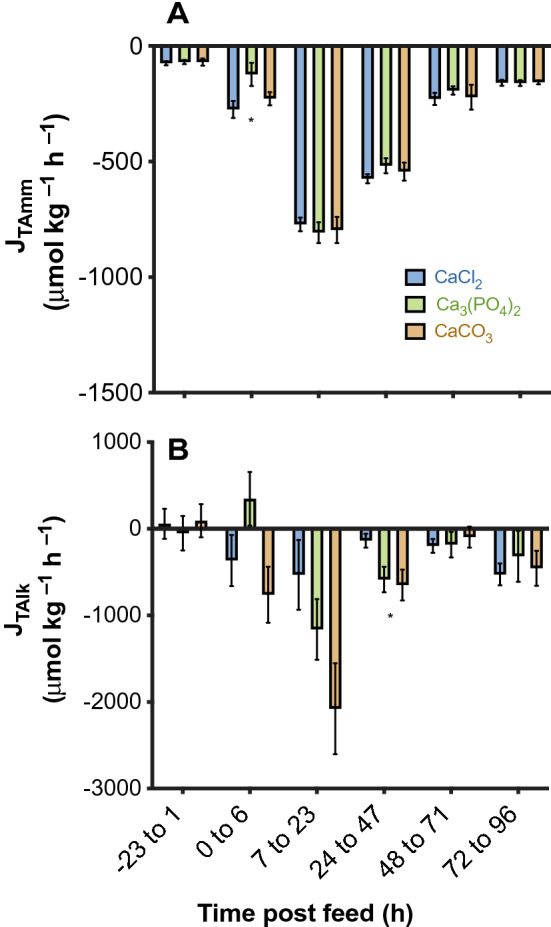
Average flux of titratable alkalinity (J_T-Alk_) (**A**) and ammonia (J_T-Amm_) (**B**) over time in juvenile rainbow trout (*Oncorhynchus mykiss*) fed a 2.5% ration of one of three experimental feeds supplemented with either Ca_3_(PO_4_)_2_, CaCO_3_ or CaCl_2_ (JTalk: *n* = 9 for Ca_3_(PO_4_)_2_ and CaCO_3_ treatments; *n* = 8 for the CaCl_2_ treatment; JTamm: *n* = 10 for all treatments). Data are expressed as means ± SE. Asterisks (*) indicates significant difference between diet groups within flux periods following a one-way ANOVA and Tukey multiple comparisons test. Significance was accepted at *P* < 0.05. For multiple comparisons within each flux period refer to Supplementary Table 3.

**Figure 4:**
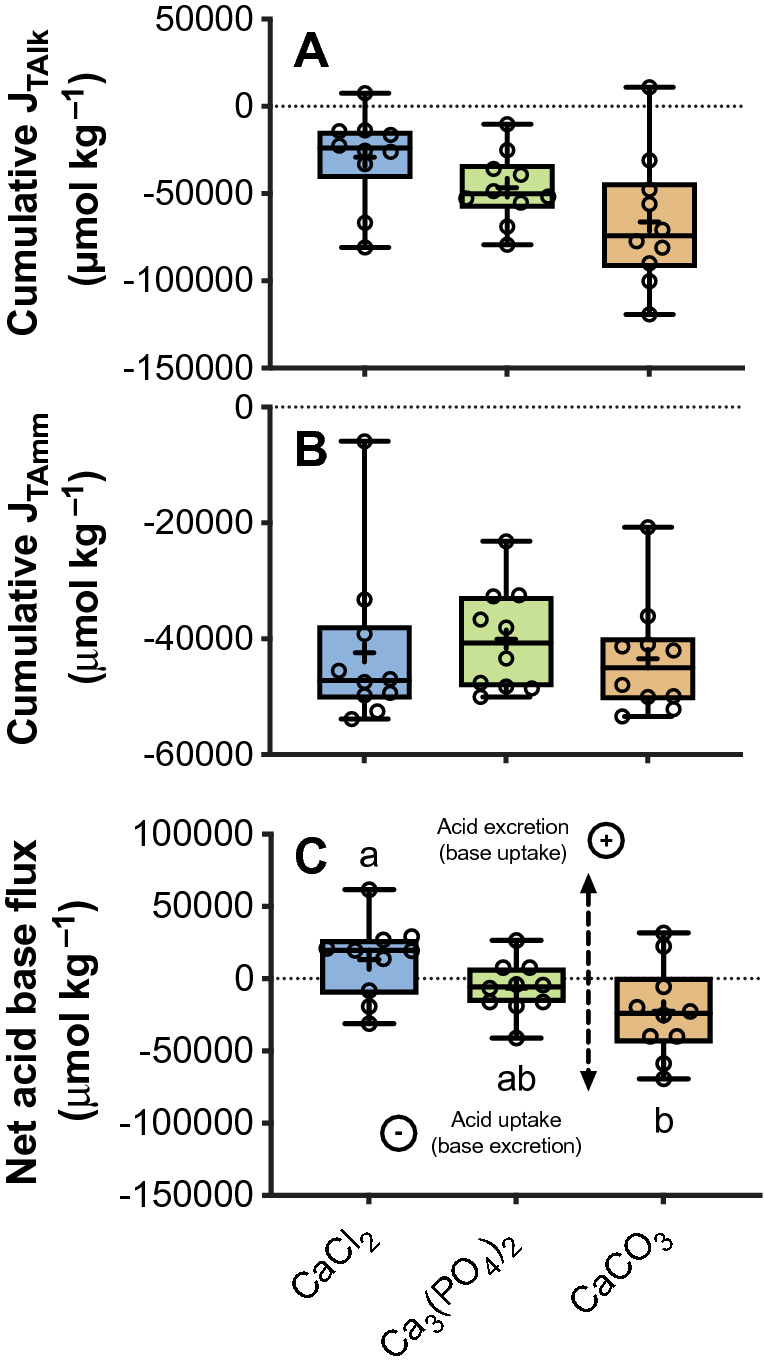
Average cumulative flux of titratable alkalinity (J_TAlk_) (**A**), ammonia (J_TAmm_) (**B**) and net acid or base (**C**) in juvenile rainbow trout (*Oncorhynchus mykiss*) fed a 2.5% ration of one of three experimental feeds supplemented with either Ca_3_(PO_4_)_2_, CaCO_3_ or CaCl_2_ (n = 10 fish). For all fluxes, positive values indicate a net base uptake (i.e. acid excretion) and negative values indicate net base excretion (i.e. acid uptake) (See panel C). Data are presented as a six number summary: minimum, first quartile (Q1), median (horizontal line), mean (+), third quartile (Q3) and the maximum. Open circles represent values from individuals. Different letters (abc) indicate statistical significance between groups following a RM one-way ANOVA and Tukey’s multiple comparisons test. Significance was accepted at *P* < 0.05.

Following feeding, all diet treatments experienced an increase in the fluxes of titratable alkalinity (J_Talk_) and ammonia (J_Tamm_). Fluxes of J_Tamm_ and J_Talk_ peaked between 7 and 23 h post feed and remained elevated above pre-feeding levels until after 48 h post feed (Fig. 3). Within each flux period there was no significant effect of diet, except at 24–47 h post feed for J_Talk_ and at 0–6 h post feed for J_Tamm_ (See Supplementary Table S3) (Fig. 3). At 0–6 h post feed J_Tamm_ was 55% greater in the CaCl_2_ diet when compared to the Ca_3_(PO_4_)_2_ diet treatment (*P* = 0.04). However, diet had no effect on the cumulative flux of T_amm_ (F_2, 18_ = 0.21, *P* = 0.81) (Fig. 4B) over the entire measurement period, and the cumulative flux of J_Tamm_ did not change with dietary buffer capacity (R^2^ < 0.01, *P* = 0.37).

In contrast, the cumulative flux of T_Alk_ significantly increased with dietary buffer capacity (R^2^ = 0.15, *P* = 0.018). The cumulative flux of T_Alk_ was almost 2.5 times greater in fish consuming the CaCO_3_ diet treatment (−66,213 ± 11,797 µmol kg^−1^) when compared to fish consuming the CaCl_2_ diet (−29,126 ± 8257 µmol kg^−1^) (Fig. 4A), however this result between groups was not significantly different (F_2, 18_ = 3.36, *P* = 0.06).

The cumulative average net acid or base equivalent flux was positive in fish consuming the CaCl_2_ diet which is indicative of a net acid excretion (or base uptake), while fish consuming the Ca_3_(PO_4_)_2_ or CaCO_3_ diets had an average negative net acid–base flux which is indicative of a net base excretion (or acid uptake). The cumulative net acid–base fluxes were 13,240 ± 8,419, −6,586 ± 5,804 and −22,753 ± 10,201 µmol kg^−1^ in fish consuming the CaCl_2_, Ca_3_(PO_4_)_2_ and CaCO_3_ diet treatments, respectively. Net base excretion significantly increased (i.e. values became more negative) with increases in dietary buffer capacity (R^2^ = 0.12, *P* = 0.03). However, multiple comparisons show that there was only a significant difference between the cumulative net acid–base flux of fish consuming the CaCl_2_ and CaCO_3_ diet treatments (F_2, 18_ = 4.21, *P* = 0.03) (Fig. 4C). In this comparison net base excretion was three times greater in fish fed the CaCO_3_ when compared to fish consuming the CaCl_2_ diet.

### The specific dynamic action after feeding

**Figure 5:**
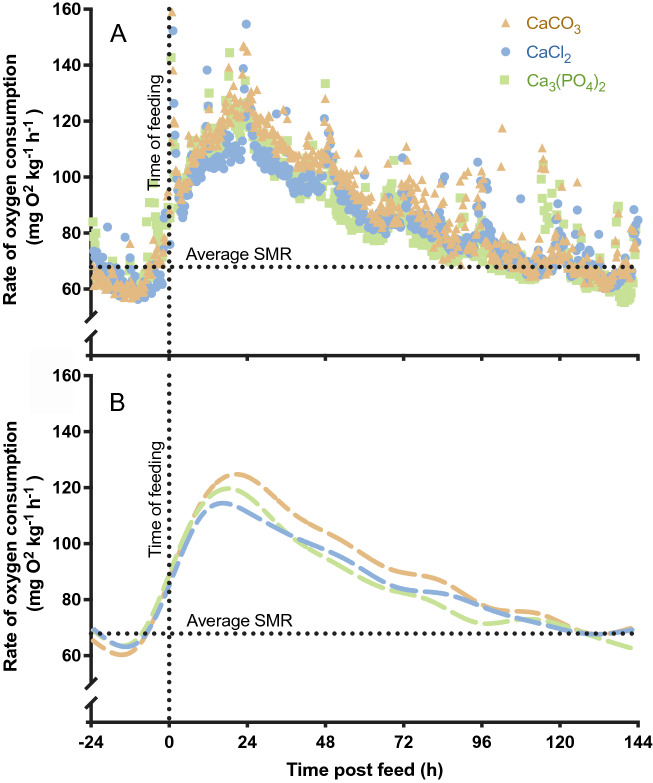
The change in oxygen consumption rate over time in rainbow trout (*Oncorhynchus mykiss*) fed voluntarily on a 2.5% ration of one of three diets supplemented with either CaCl_2_, CaCO_3_ or Ca_3_(PO_4_)_2_ (n = 8). Data are presented as mean values for each respirometry cycle time point (A) and smoothed pattern over time using a cubic squared spline (B). The horizontal line represents the average resting (pre-feeding) oxygen consumption of all treatments 24 h prior to feed. The vertical dotted line represents time of feeding.

**Figure 6:**
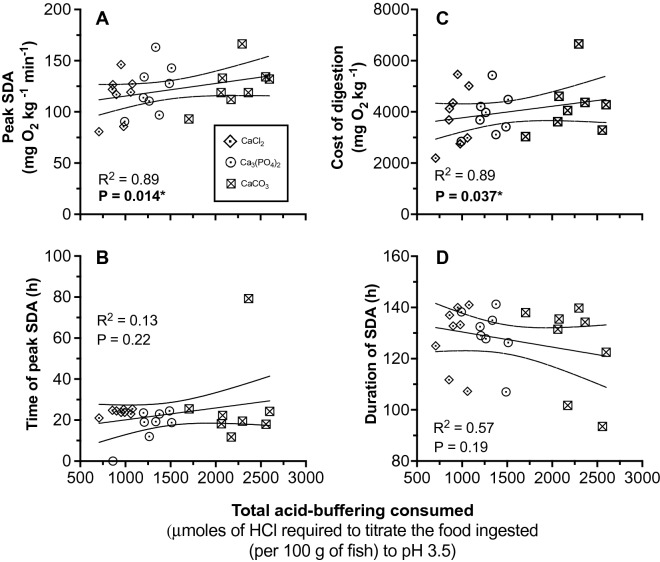
Change in the peak SDA (MO_2_) (**A**) time of peak (**B**), total energetic cost of digestion (SDA; **C**) and total duration of the SDA (**D**) in juvenile rainbow trout (*Oncorhynchus mykiss*) fed voluntarily on a 2.5% ration of one of three experimental feeds (n = 8) of varying buffer capacity. The ranked order (and relative magnitude) of the dietary acid-buffering capacity of each diet was CaCO_3_ > Ca_3_(PO_4_)_2_ > CaCl_2_ (2.4 > 1.4 > 1). Solid line represents the regression line while the dotted line represents the 95% confidence interval (CI). Significance was accepted at *P* < 0.05 following a linear mixed effects model. Each data point represents values from individuals.

All fish experienced an increase in the rate of oxygen consumption following feeding that peaked between 19 and 21 h and lasted between 125 and 130 h after meal ingestion (Fig. 5, Table 1). The peak in postprandial oxygen consumption was greatest in fish consuming the CaCO_3_ diet (127 ± 7 mg O_2_ min^−1^ kg^−1^), lowest in the CaCl_2_ group (115 ± 7 mg O_2_ min^−1^ kg^−1^) and intermediate in the Ca_3_(PO_4_)_2_ group (122 ± 8 mg O_2_ kg^−1^ min^−1^) (F_2, 14_ = 4.21, *P* = 0.04; R^2^ = 0.89, *P* = 0.014) (Table 1, Fig. 6A). Multiple comparisons show that peak oxygen consumption significantly differed between the CaCl_2_ and CaCO_3_ groups only (*P* = 0.03) (Table 1).The timing of this peak was similar across diet treatments (F_2, 13_ = 0.05, *P* = 0.94; R^2^ = 0.13, *P* = 0.22) (Fig. 6B, Table 1).

**Table 1:** Summary of SDA responses in juvenile rainbow trout (*Oncorhynchus mykiss*) fed a 2.5% ration of a diet supplemented with either CaCl_2_, Ca_3_(PO_4_)_2_ or CaCO_3_ (n = 8 animals each consuming all three diets).

	Diet (Buffer capacity: μmol HCl required to titrate 1 g of material to pH 3.5)		
	CaCl_2_ Control (228 μmol HCl)	Ca_3_(PO_4_)_2_ Bone (320 μmol HCl)	CaCO_3_ Shell (550 μmol HCl)
Magnitude (mg O_2_ kg^−1^) (Total cost of digestion)	3822 ± 401^a^	3896 ± 293^a^	4238 ± 396^a^
Duration (h)	128.50 ± 4.52^a^	129.63 ± 3.72^a^	123.61 ± 6.21^a^
Initial peak MO_2_ (mg O_2_ kg^−1^ h^−1^)	**115.57 ± 7.75**^**a**^	122.49 ± 8.58^ab^	**126.07 ± 7.51**^**b**^
Time to initial peak (h)	20.97 ± 3.04^a^	20.47 ± 1.47^a^	20.97 ± 7.57^a^
SDA coefficient (%)	9.30 ± 0.98^a^	9.48 ± 0.71^a^	10.32 ± 0.96^a^
SDA (kJ kg^−1^)	53.52 ± 5.62^a^	54.55 ± 4.10^a^	59.34 ± 5.54^a^
SDA scope	2.12 ± 0.09^a^	2.22 ± 0.08^a^	2.27 ± 0.09^a^

Lowercase letters and bold text indicate significant differences between diets at *P* < 0.05 following a repeated measures one-way ANOVA (RM- ANOVA) and Tukey multiple comparisons test.

Data are presented as mean ± SE.

The regression analysis showed that the total cost of digestion (the SDA) significantly increased with dietary buffer capacity (R^2^ = 0.89, *P* = 0.04) (Fig. 6C). Fish consuming the non-buffering CaCl_2_ diet had the lowest cost of digestion at 3,822 ± 401 mg O_2_ kg^−1^ body mass (53.5 ± 5.6 kJ kg^−1^) while fish consuming the CaCO_3_ diet had the greatest cost of digestion at 4,327 ± 395 mg O_2_ kg^−1^ (59.3 ± 5.5 kJ kg^−1^), i.e. ~ 11% higher. However, following a RM-one way ANOVA, there was no significant difference between diet groups (F_2, 14_ = 2.11, *P* = 0.16) (Table 1). Similarly, the SDA coefficient (%) significantly increased with dietary buffering (R^2^ = 0.89, *P* = 0.04), but the RM-ANOVA and multiple comparisons showed no difference in the SDA coefficient between diet groups (F_2, 14_ = 2.11, *P* = 0.16).

The duration of the SDA (time when metabolic rate is no longer significantly different from SMR) did not vary with dietary buffering (R^2^ = 0.57, *P* = 0.19) or between groups (F_2, 14_ = 0.63, *P* = 0.54) (Fig. 6D, Table 1). Rates of oxygen consumption returned to pre-feeding levels for all diet treatments within the same 5 h window (between 125–130 h post feed).

### Trout growth efficiency

**Figure 7:**
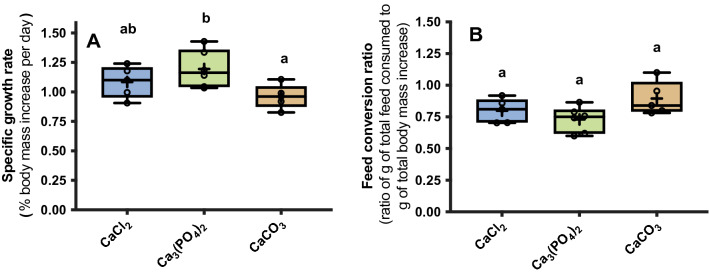
The average specific growth rate (SGR) (A) and feed conversion ratio (FCR) (B) of juvenile rainbow trout (*Oncorhynchus mykiss*) fed a daily 1% ration of three experimental feeds (*N* = 5 for CaCl_2_ and CaCO_3_ treatments; *n* = 6 for Ca_3_(PO_4_)_2_ treatment) over a 21 day period. Data are presented as a six number summary: minimum, first quartile (Q1), median (horizontal line), mean (+), third quartile (Q3) and the maximum. Open circles represent values from each individual. Different letters (abc) indicate statistical significance between groups following a one-way ANOVA and Tukey’s multiple comparisons test. Significance was accepted at *P* < 0.05.

The feed conversion ratio (FCR) ranged between 0.72 ± 0.04 in fish consuming the Ca_3_(PO_4_)_2_ diet and 0.89 ± 0.06 in fish consuming the CaCO_3_ diet. Similarly, the specific growth rate (SGR) was greatest in the Ca_3_(PO_4_)_2_ diet group (1.19 ± 0.06%), lowest in the CaCO_3_ diet group (0.96 ± 0.05%) and intermediate in the CaCl_2_ diet group (1.09 ± 0.06%). Dietary buffer capacity had no effect on the FCR or SGR (FCR: R^2^ = 0.15, *P* = 0.1; SGR: R^2^ = 0.16, *P* = 0.1). Similarly, the FCR did not differ between any treatment groups (F_2, 13_ = 3.09; *P* = 0.08) (Fig. 7B). However, there was a significant difference in the SGR of fish fed each diet (F_2, 13_ = 4.0, *P* = 0.04). Fish consuming the highest buffered CaCO_3_ feed had a significantly lower SGR than the Ca_3_(PO_4_)_2_ diet treatment (*P* = 0.034) but not the CaCl_2_ diet treatment (*P* = 0.35) (Fig. 7A). Similarly, there was no significant difference in SGR between the CaCl_2_ and Ca_3_(PO_4_)_2_ treatments (*P* = 0.39).

## Discussion

This study shows that the acid buffering properties of food, independent of caloric or macronutrient content, can influence the magnitude and/or peak in energetic and physiological costs associated with digestion in fish. We show that this is due to the impact of buffering minerals on the physiological processes associated with digestion. Increasing the buffering capacity of a pelleted fish diet with calcium salts known to be present in bone (Ca_3_(PO_4_)_2_) and shell (CaCO_3_) increased gastric pH, the alkaline tide, base excretion, and the SDA following feeding. Unexpectedly, despite enhanced energetic and physiological costs, increasing the buffer capacity of fish diets did not influence the duration of the SDA or fish growth efficiency over a 21 day growth trial.

Increases in dietary buffer capacity led to an increase in stomach chyme pH with the CaCO_3_ diet generating the most alkaline stomach pH compared to all other diet treatments. The need for greater gastric acid secretion in the two diets with enhanced buffering (CaCO_3_ and Ca_3_(PO_4_)_2_) coincided with a significant increase in blood HCO_3_^−^ at 24 h post feed and greater cumulative net base excretion following feeding. More specifically, following meal consumption, fish consuming the highly buffered CaCO_3_ diet had a net base excretion almost three times greater than fish consuming the non-buffering CaCl_2_ diet. This suggests that fish consuming this most buffered diet required the greatest gastric acid secretion in order to complete digestion, and therefore experienced the greatest increase in blood HCO_3_^−^ and net base excretion following feeding.

It is likely that the high rates of base excretion during the first 24 h after feeding allowed all fish to recover from the post prandial alkaline tide by 48 h after meal consumption. Cooper and Wilson (2008)^19^ show that blood pH and HCO_3_^−^ concentration following the consumption of a 1% ration in rainbow trout was greatest 6–24 h after feeding, and had returned to pre-feeding levels by 48 h post feed. Wood et al. (2005) also reported a large efflux of basic equivalents to the external water by the spiny dogfish (*Squalus acanthias*) after voluntary feeding at these same time points. In the current study all fish were able to recover from the post prandial alkaline tide by at least 48 h after feeding, but the CaCO_3_ group actively compensated for a significantly greater HCO_3_^−^ base load via greater cumulative net base flux to the water.

Here we also provide evidence to suggest that the intestine may play a greater role in recovering from the post prandial alkaline tide than previously thought. Previous studies delineate the role of the fish gill and kidney in recovering from the alkaline tide^25,26^, with the majority of research suggesting the gills may contribute more so than the kidney^27^. In the present study we observed large concentrations of intestinal HCO_3_^−^ in all fish and diet treatments at 48 h post feed. Across all three diets, intestinal chyme pH ranged between 8.12 ± 0.13 and 8.26 ± 0.18 in the CaCl_2_ and Ca_3_(PO_4_)_2_ diet treatments, while the concentration of intestinal chyme HCO_3_^−^ ranged between 47 and 60 mM in the two more buffered diet treatments. Interestingly these values are more similar to that of a marine fish than a freshwater fish when not feeding. It is suspected that some of the blood load of HCO_3_^−^ resulting from gastric acid secretion was transported to the intestinal lumen and excreted to contribute to the recovery of internal acid–base and ion balance. This finding is supported by two separate studies on marine teleosts, European flounder (*Platichythus flesus*)^28^ and gulf toadfish (*Opsanus beta*)^29^. These studies observed elevated levels of HCO_3_^−^ in the intestine following the consumption of a meal and concluded that the intestine functions in contributing to recovery from a metabolic alkalosis^28,29^. The current study provides further evidence to support the role of the intestine in post prandial base excretion in fish, and the first record of HCO_3_^−^ secretion to the intestine by a freshwater-acclimated euryhaline species. However, despite working on the same species as the present study, Cooper and Wilson (2008)^19^ concluded that the intestine did not contribute to the recovery from the alkaline tide and suggested this may be due to the delayed expression of the necessary transporters required for intestinal HCO_3_^−^ secretion. Although, in their study fish were fed on a substantially smaller ration of feed (1%) than in the present study (2.5%). A smaller ration would reduce the requirement for gastric acid secretion, resulting in a smaller alkaline tide and lesser need to excrete excess blood HCO_3_^−^. In the present study the 2.5 times greater ration would mean that even the non-buffered diet would have resulted in a proportionally higher rate of gastric acid secretion and presumably bicarbonate loading in the blood which would require excretion. This may contribute to the intriguingly higher levels of intestinal bicarbonate in the present study. We also speculate that the ingestion of equimolar amounts of additional calcium in all three diets could have generated a phenotypic change in intestinal function in freshwater rainbow trout. High levels of calcium in ingested seawater are thought to be a key stimulus of the intestinal bicarbonate secretion process when marine teleosts drink seawater for osmoregulation^30,31^. Indeed calcium-sensing receptors are believed to be a key sensor that induces osmoregulatory processes generally in fish^32^. It is therefore possible that the extra calcium in all the diets of our freshwater trout played a similar stimulatory role and facilitated the intestinal secretion of excess blood HCO_3_^−^. In doing so this would presumably contribute to a speedier than otherwise recovery of internal acid–base balance following the post prandial alkaline tide (i.e. earlier than if the extra calcium was not present in the diet). These findings provide interesting new avenues of investigation for the study of gastrointestinal physiology in freshwater fishes.

We propose that these enhanced physiological costs associated with more buffered diets led to the significant increase in the peak oxygen consumption and the total energetic cost of digestion in the current study. These results are consistent with the hypothesis that increasing the requirement for gastric acid secretion and recovering from the associated blood alkalosis is energetically expensive in fish. Previous research has highlighted that gastric HCl secretion is a relatively expensive process^14,15,17^. In the recent study from Goodrich et al. (2022)^12^, acidifying a commercial fish feed led to a reduction in the energy cost of digestion by almost half in juvenile barramundi (*Lates calcarifer*) when compared to fish consuming a control non-acidified feed. The apparent energetic costs of gastric acid secretion during digestion are also corroborated by a multitude of studies in snakes. Secor (2003)^33^, stated that as much as 55% of the SDA response could be linked to gastric processes in the Burmese python (*Python molurus)*. Similarly, in the garter snake (*Thamnopis sirtalis*), the consumption of vertebrate prey led to a larger SDA than when compared to softer body prey^34^. Here, the increased buffer capacity of a vertebrate prey item would have increased gastric handling and likely contributed to the increase in SDA. In a study on the Atlantic cod (*Gadus morhua*) gastric emptying (the process of chyme release from the stomach to the intestine) was significantly delayed in prey types with increased ash and carbonate content^35,36^. The authors theorised that the elevated ash and carbonate content of some prey items would have increased the buffering capacity of the meal, thereby elevating stomach pH and prolonging gastric handling time. Likewise, we expect that the increased requirement for HCl secretion in fish fed the highly buffered CaCO_3_ or ‘shell’ diet would have increased energy demands and contributed to the observed increase in energy expenditure during digestion.

Alongside the energetic costs associated with gastric acid secretion, it’s likely that fish consuming the more highly buffered “shell” and “bone” diets would have experienced additional physiological costs from the enhanced base excretion associated with a greater alkaline tide. In the current study, we observed a greater blood HCO_3_^−^ concentration at 24 h post feed in fish fed the CaCO_3_ and Ca_3_(PO_4_)_2_ diets. As discussed previously, this was associated with a greater net base excretion to the external environment when compared to fish fed the non-buffering CaCl_2_ meal. Despite what we know about the energetic costs associated with gastric acid secretion, much less is known about the energetic costs associated with other tissues (gills, kidney, intestine) in recovering from metabolic acid–base disturbances like the alkaline tide in fish. However, it is widely accepted that acid–base and ion regulation will incur some energetic cost, although quantifying these costs has been difficult. Some studies have suggested that ion regulation in response to living in either hypo- or hyper-saline environments (i.e. freshwater or seawater) could account for as much as 30% of an animals standard metabolic rate^37^. In addition to a greater cost at the gills for fish fed a buffered diet, it is likely that an enhanced feeding-induced metabolic alkalosis also has consequences for oxygen transport via haemoglobin^38^. A rise in blood pH increases haemoglobin’s affinity for oxygen^39^ which would enhance oxygen uptake at the gills^39,40^ but could impair oxygen delivery to tissues. In theory this should present a significant challenge for fish during digestion, due to the increased demand for oxygen necessary to breakdown and assimilate a meal (i.e., the SDA). In turn highly buffered diets may not only enhance the magnitude of the alkaline tide but could also lead to energetic and physiological consequences for blood oxygen delivery via haemoglobin. This could create an additional challenge to the respiratory transport systems during digestion, which would require further physiological responses potentially with additional costs; e.g. if it requires a compensatory increase in cardiac output to overcome. Therefore, future studies may wish to assess the affinity of haemoglobin for oxygen during digestion in fish fed diets with varying buffer capacity as a tool to manipulate this particular respiratory challenge.

Interestingly, the cumulative costs associated with the digestion of a buffered feed did not influence fish growth over 21 days in the way we expected. Despite a greater physiological and energetic burden, there was no relationship between fish growth and dietary buffering. These results show that a reduction in the SDA will not always coincide with greater growth efficiency. Similar growth results were observed by Goodrich et al. (2022) following chronic exposure to a feed that significantly reduced the SDA. In the current study, when compared to the Ca_3_(PO_4_)_2_ treatment group, fish consuming the CaCO_3_ diet grew significantly less. It is expected that the slight but not significant increase in growth of fish consuming the Ca_3_(PO_4_)_2_ diet when compared to the CaCl_2_ diet could be attributed to greater bone growth. Phosphate supplementation is a common practice in aquaculture due to its pivotal role in intracellular processes such as muscle contraction, but also its key role in bone growth (the most abundant use of phosphate in the body)^41,42^. Adebayo and Akinwumi (2015)^43^ found significantly greater concentrations of phosphorous in the carcass of *Clarias gariepinus* fingerlings consuming a diet supplemented with bone meal. This finding also coincided with a greater specific growth rate in this treatment group^43^. In contrast, we predict that the slight but not significant decrease in fish growth and increase in the feed conversion ratio of fish consuming the CaCO_3_ diet when compared to the CaCl_2_ diet can be in part attributed to the greater costs associated with the digestion of a highly buffered feed, but without the benefit of additional phosphate to support greater bone growth. As well, the growth trial in the current study was conducted on a daily 1% ration of feed, which was much less than the 2.5% ration used to conduct the SDA trials. It is likely that growth differences could exist if fish were fed more each day or if fish were fed diets with greater differences in dietary buffering, or if the trial was extended to a longer period of time. However, it is also possible that stomach acidification and its associated responses may not contribute as greatly to the SDA in rainbow trout, or that reductions in the SDA do not always coincide with greater growth efficiency. Future studies could assess the growth effects of a buffering diet on smaller fish for a longer time period and over a greater range of dietary buffering to determine if naturally occurring buffering minerals have the capacity to affect growth through impacts on the costs associated with gut digestion.

The results presented in this study have important implication for the design of fish feeds in aquaculture. Meeting the needs of the growing human population has already led to the over exploitation of most wild fisheries and driven the exponential growth of and requirement for intensive aquaculture. Recognising this demand, the Food and Agriculture Organisation (FAO) of the United Nations has called for aquaculture to double seafood production by 2050. Improving the efficiency and sustainability of intensive finfish aquaculture will be key to address this aim. Fish feeds provide an avenue to directly influence the efficiency and sustainability of aquaculture. Currently, many fish feeds have buffering properties (ability to resist change in pH) that could have physiological and energetic consequences for digestion in fish. Reported previously by Goodrich et al. (2022), the raw ingredients used by feed manufacturers can vary substantially in their buffering properties. Depending on demand and product availability, different types or combinations of raw materials can be used to produce the exact same fish feed product^12^. This can cause variation in the total buffering properties between batches of the same feed. Based on the results from the current study, variation in dietary buffering could unintentionally influence the physiological and energetic costs associated with digestion in aquacultured fish. Designing feeds with raw materials that reduce the physiological and energetic burden placed on the animal could provide benefits to the production of fish in aquaculture.

In wild fish additional energy costs associated with the digestion of more highly buffered diets could influence prey choice. Understanding the feeding responses, patterns and preferences of fish in the wild helps to understand fundamental ecological principles, can inform the management of aquatic environments and be used to make predictions about current and future impacts of climate change on fish communities. The results presented here show how the non-nutritive parts of a diet can influence energetic costs associated with digestion. Interestingly, we show that increases in dietary buffer capacity can increase the peak oxygen consumption associated with the SDA. This is an effect of dietary buffering that was also observed in Goodrich et al. 2022^12^. In that study fish fed on an acidified diet experienced a ~ 18% reduction in the secondary peak of the SDA when compared to a more buffered control diet. This could have important implications for the feeding responses of fish experiencing limitations to their aerobic scope, such as occurs during environmental warming. A recent review has suggested that the quantity of food consumed by fish will depend on the available postprandial residual aerobic scope (PRAS) during digestion^44^. Aerobic scope describes the difference between the maximum and resting rates of oxygen consumption^45^, while PRAS describes the available aerobic scope during the peak of the SDA. PRAS governs the scope available for activities outside of digestion like swimming and avoiding predation. In elevated temperatures the SDA is temporally compressed leading to an increase in peak oxygen consumption during digestion and a reduced post residual aerobic scope. In response to this, it is hypothesised that fish reduce feed intake at elevated temperatures in order to protect their post residual aerobic scope and maximise the scope available for other activities. This is a clear example of how physiology can influence the feeding behaviour of fishes. Given the impact of dietary buffering on peak oxygen consumption during digestion, it’s possible fish may select for prey that not only maximises their net energy gain, but simultaneously best protects their aerobic scope. We observed no statistically significant effect of dietary buffering on fish growth, but this was using a small ration size (1% / day) and short growth period (3 weeks). However, when faced with a physiological challenge such as warming or hypoxia, it would be logical for fish to preferentially select for prey items that maximise PRAS. This could influence the feeding responses of fishes in the wild and lead to shifts in prey selection as a result of anthropogenic warming. To investigate this idea, future studies may wish to investigate the relationship between dietary buffering, warming, PRAS and prey selection.

In predatory fish species with flexible prey choices, differences in prey selection may be more pronounced. For example, the lionfish *Pteroid volitans* is an invasive species that has occupied reefs in the Western Atlantic Ocean and continues to spread throughout the Caribbean and the East Coast of the United States. This predatory species is responsible for a drastic decline in the diversity of reef fishes from these regions^46–48^. The invasive ability of the lionfish has been helped by its lack of natural predators, opportunistic lifestyle and ability to consume most prey types. Research into the foraging ecology of lionfish found that stomach contents consisted of 78% teleosts and 14% crustaceans, suggesting a preference for bony fishes over shelled crustaceans^49^. The potential energy gain and reduced impact on PRAS during digestion of a meal of fish (with a calcium phosphate-based skeleton) versus crustaceans (with a calcium carbonate-based shell) could in part help to explain why lionfish have a preference towards bony fishes. To investigate these ideas, future research could assess the relationships that exist between dietary buffering, the SDA and the prey choices of predatory fish. Indeed, such studies would provide great insights into predator prey interactions and the feeding ecology of wild fishes.

## Conclusion and future directions

Most nutritional-focused studies measuring the SDA response in fish have worked to understand how the nutritional composition of a meal (lipid, carbohydrate, protein) influences the energy cost of digestion and fish growth. For the first time we show that the non-nutritive components of a diet can also have implications on energy use during digestion in fish. We demonstrate that the digestion of diets with elevated buffering can enhance requirement for acid secretion to the stomach, increase the alkaline tide and net base excretion, cost fish more to digest, but don’t influence fish growth efficiency. Given the findings from this study and current known gaps in knowledge, we suggest a number of future directions for research:

1. Investigate the relationship between dietary buffering, the SDA and growth efficiency using a larger daily feed ration, diets with a greater difference in dietary buffering and longer growth trial period.
2. Assess the effect of feeding, digestion and the alkaline tide on the affinity of haemoglobin to bind oxygen in fish, i.e. the blood P_50_ value.
3. Investigate the relationships between temperature and or hypoxia on the post-residual aerobic scope (PRAS), SDA, prey choice and feed intake in fishes.
4. Test the application of an aquaculture diet made from raw materials with reduced dietary buffering.
5. In wild fish, determine whether dietary buffering and the cost of digestion can influence prey choice and/or preferences.

## Materials and methods

### Animal ethics

All experiments were conducted under the UK Home Office licence P88687E07 and with approval from the University of Exeter Ethics Committee.

### Fish husbandry

Juvenile rainbow trout (*Oncorhynchus mykiss*) (*n* = 42; body mass: 159.9 ± 5.2 g), were obtained from Houghton Spring Fish Farm (Dorset, UK) and housed in the Aquatic Research Centre at the University of Exeter (UK). Before transfer to individual experimental chambers, all fish were housed across two 400 L tanks (n = 21 per tank) supplied with recirculated fresh water for 14 days. During this 14 day acclimation period, fish were maintained at 15 °C and fed on a 1% ration of commercial trout feed (Aller platinum 4.5 mm (Aller AQUA ©) three times a week. Prior to experimentation, fish were fasted for seven days.

### Acid buffering diets

Diets were prepared by adding one of three calcium-based salts, CaCO_3_, Ca_3_(PO_4_)_2_ or CaCl_2_ (as non-buffering control) with isomolar quantities of calcium to a commercial trout pelleted diet (Skretting 4.5 mm Horizon, Skretting, UK). The quantities of these salts used were designed to mimic the calcium content of the skeletal component of crustacean or bony fish prey.

Cameron (1985)^50^ estimated that the bone of teleost fish represents 16.3% of whole-body mass (and therefore soft tissue represents 83.7%). However, bone is not just calcium phosphate, but includes numerous organic components as well as water content. By comparing titrations of pure calcium phosphate salt and samples of ground-up teleost (rainbow trout) bone, we established that it required 10.25 times less calcium phosphate salt to achieve the same acid-buffering capacity as that of an equal mass of bone. We therefore created a diet that was supplemented with 1.9 g calcium phosphate for every 100 g of trout pellets (i.e. [16.3 g ÷ 10.25] x [100 ÷ 83.7 g] = 1.9 g), in order to match the bone content of calcium phosphate typically found in fish prey as a proportion of the soft tissue mass. This amounted to 18.4 mmoles of calcium phosphate salt (Ca_3_(PO_4_)_2_; M.W. = 310.2) per 100 g of trout pellets. For the two other diets we aimed to maintain the same molar amount of calcium cation added whilst varying the anionic component of the salt added. So, for the unbuffered version of the diet 2.7 g of calcium chloride (CaCl_2_.2H_2_O; M.W. = 147.0) was added, whilst for the calcium carbonate (CaCO_3_; M.W. = 100.0) buffered diet 1.84 g was added, per 100 g of trout pellets.

To form each diet, 100 g of Skretting 4.5 mm Horizon trout pellets were ground to a fine powder using a pestle and mortar. Following grinding, 1.9, 1.84 and 2.7 g of Ca_3_(PO_4_)_2_, CaCO_3_ and CaCl_2_ were added to the ground pellet and mixed. Then, 70 ml of ultrapure water was added to the dry material to form a paste. This paste was pressed into commercial 4 mm moulds, removed and dried at 70 °C for 24 h. An acid titration test was conducted to ensure that diets remained representative of the buffer capacity of prey and each calcium salt. For this test, 60 ml of ultrapure water were added to 1 g of each experimental diet and titrated down to pH 3.5 using 0.05 mol L^−1^ HCl. The CaCl_2_ diet treatment required 4.56 ml of the acid which was only slightly less than the 6.4 ml required to titrate the Ca_3_(PO_4_)_2_ diet. In contrast it took almost double the amount of acid (11 ml) to titrate the CaCO_3_ diet. In molar terms it took 228, 320 and 550 µmoles of HCl to titrate 1 g of the CaCl_2_, Ca_3_(PO_4_)_2_ and CaCO_3_ feeds to pH 3.5, respectively. To calculate the total acid-buffering consumed, the buffer capacity (per g of food) was multiplied by the actual ration ingested for each individual. Based on manufacturer details each diet had a gross energy of 23 kJ per gram of feed.

### Acid secretion in the stomach and the blood alkaline tide

To investigate the effect of dietary buffer capacity on the blood acid–base chemistry (alkaline tide) and gut secretions, blood and gut samples were taken from fish to determine blood gas and acid–base balance and haematology variables of fish fed each experimental diet. Fish were fasted for 7 days and then fed a 2.5% ration of one of three experimental feeds. Diet was randomly allocated to each individual (n = 6 per diet). At 24 and 48 h following meal ingestion fish were anesthetised using benzocaine (100 mg l^−1^). Once fish had lost equilibrium and were un-responsive to a tail pinch, fish were transferred to a gill irrigation system dosed with a lower concentration of benzocaine (75 mg l^−1^). Fish were placed upside down within the irrigation chamber so that the head was fully submerged, and the entire gill basket covered. A micro pump was used to artificially ventilate the gills via a tube placed into the fish mouth. This allowed for the continuous ventilation of fish gills and ensured there was no build-up of CO_2_ or lactic acid during blood sampling that could unintentionally affect blood acid–base status. Blood was then drawn into a sodium-heparinised syringe via caudal puncture. Fish were then euthanased via pithing and dissected to collect stomach and intestinal contents. Gut samples were centrifuged to isolate gastric and intestinal juices.

Blood and gastric pH were measured using an Accumet CP-620-96 MicroProbe (Accumet Engineering Corporation, USA) connected to a Hanna HI 8424 m (Hanna Instruments, Woonsocket, Rhode Island, USA). Whole blood PO_2_ was measured using a Strathkelvin 1302 electrode, housed within a thermostatted glass chamber (Strathkelvin), and connected to Strathkelvin 781 m (Strathkelvin Instruments Ltd., Scotland)^51^. Blood was drawn into three micro-haematocrit tubes (Hawksley) via capillary action and anaerobically sealed using Hawksley Critaseal Wax Sealant, then centrifuged (Hawksley microhaematocrit centrifuge, 10,000 rpm for 2 min) and then used to record haematocrit and held on ice before using the plasma. Plasma and intestinal total CO_2_ was then measured using a Mettler Toledo 965 carbon dioxide analyser and together with blood and intestinal pH measurements was used to calculate plasma and intestinal HCO_3_^−^ and PCO_2_ by rearranging the Henderson–Hasselbalch equation and using values for solubility and pK_app_ from Boutilier et al. (1985)^52^.

### Net acid–base fluxes to the external water

The effect of diet on the net flux of acid–base relevant ions to the external water was measured in a separate subset of juvenile rainbow trout (n = 10, 161.8 ± 6.9 g). Prior to measurements fish were weighed and transferred to individual 25 L chambers supplied with recirculated freshwater maintained at 15 °C. Following a 3-week acclimation period, fish were fed weekly on a 2.5% ration of one of three experimental feeds, with diet order randomised to each individual (See Supplementary Table 4). Initial and final water samples were taken from each chamber over six flux periods each week for three weeks (−23 to 1 (fasted), 0–6, 7–23, 24–47, 48–71 and 72–96 h post feed). Water inflow to each chamber was turned off during each flux period whilst aeration was maintained. Following the final measurement from each flux period, tanks were flushed with dechlorinated freshwater for 60 min so to ensure solid faeces and dissolved waste products (e.g., ammonia) were removed.

Total ammonia was measured in triplicate on 200 µL water samples using the colourimetric salicylate-based method adapted from Cooper and Wilson (2008)^19^ and Verdouw et al. (1978)^53^ and the Infinite 200 PRO microplate reader (Tecan Trading AG Switzerland ©). Titratable alkalinity was measured in 20 ml water samples using an auto-titrator with autosampler (Metrohm 907 Titrando with 815 Robotic USB Autosampler XL) running double titrations with 0.02 mol l^−1^ of HCl and 0.005 mol l^−1^ NaOH. The double titration method calculates titratable alkalinity based on the difference in HCl required to titrate each water sample down to pH 3.9 and the amount of NaOH required to bring the sample back to the starting pH. During the titration, the sample is continuously bubbled or ‘purged’ with the inert gas N_2_ to remove any CO_2_. The net fluxes of titratable alkalinity (*J*_Talk_) and total ammonia (*J*_Tamm_) were calculated using the following equation from Cooper and Wilson 2008:

1 $${J}_{\mathrm{net}}\mathrm{X}=\frac{[\left(\left[{\mathrm{X}]}_{i}-{\left[\mathrm{X}\right]}_{\mathrm{f}}\right) \times V\right]}{(M \times t)}$$

where X_i_ and X_f_ are the initial and final ion concentration in each tank (μmol l^−1^) from each flux period, *V* is the tank volume (L), *M* is the animal mass (kg) and *t* is the flux duration (h).

The net acid–base flux was calculated as the difference between the flux of titratable alkalinity (*J*_Talk_) and the flux of total ammonia (*J*_Tamm_).

### Measuring the SDA

Intermittent flow-through respirometry was used to determine the rate of oxygen consumption (MO_2_) by juvenile rainbow trout fed voluntarily on a 2.5% ration of three experimental feeds. Prior to measurements, juvenile rainbow trout (*n* = 8, 162.2 ± 7.5 g) were weighed and transferred to individual 25 L chambers supplied with recirculated freshwater at 15 °C for 3 weeks. During this acclimation period, fish were fed weekly on a 2.5% ration of Skretting 4.5 mm Horizon trout pellets (Skretting UK). Following this acclimation period, measurements were conducted after 7 days of fasting. Each fish was fed once per week on all three diets over a 3-week period, with diet order randomised for each individual.

During experimentation, fresh water was supplied continuously to two aerated 160 L sumps each fitted with a ballcock valve and overflow. Aerated freshwater was then pumped from the sump to the eight respirometry chambers in a loop for the duration of the testing period. Water within each fish chamber was continuously mixed using a submerged mini-pump (WP300; Tetra Werke, Melle, Germany). During measurements, water inflow to each chamber was shut off and the decline in O_2_ was recorded by PO_2_ OxyGuard Mini Probe (OxyGuard ® International, Denmark) connected directly to the mini-pump. Oxygen partial pressure values were logged continuously by Pyro Oxygen Logger software (Pyroscience GmBH, Germany) which interfaced with a respirometry software package (AquaResp3: aquaresp.com, see Svendsen et al. 2016 ^54^) to instantaneously convert *P*O_2_ into O_2_ content and calculate the rate of oxygen consumption (MO_2_, mg O_2_ kg^−1^ body mass h^−1^) based on the fish body mass in kg (m), chamber water volume in L after discounting the fish body volume (V_resp_), and the slope (s) of the decline in oxygen concentration (kPa O_2_ h^−1^) versus time using the following equation from Svendsen et al. (2016)^54^:

$${MO}_{2}= {sV}_{Resp}{\alpha m}^{-1}$$

where:

$$s= \frac{{O}_{2}\, initial- {O}_{2}\, final}{time\, initial-time\, final}$$

Following each closed measurement period, the chamber was automatically flushed with freshwater from the aerated sumps by two AquaMedic Ocean Runner pumps (Aqua Medic, Ocean Runner 6500). The length of the flush and measurement periods was controlled by two USB- 4 Cleware switches (Cleware GmbH, Germany) which were also interfaced with the AquaResp software to ensure that the partial pressure of oxygen (*P*O_2_) within the respirometry chambers never fell below 90% of the starting value. This meant that the measurement period of 15 min was followed by a flushing period of 2 min and a wait time of 60 s.

Prior to feeding a baseline 24 h period of standard metabolic rate (SMR) was recorded. The mean SMR of each individual was calculated using the R package ‘fishMO2’ and the ‘calcSMR’ function. Following Chabot et al. (2016)^55^, the coefficient of variation (CVmlnd) was used to determine whether the mean of the lowest normal distribution (MLND) or the quantile method (*P* = 0.2) was used to estimate SMR for each individual. Following the SMR measurement, fish voluntarily fed on a 2.5% ration of experimental feed and MO_2_ recorded continuously for six days. This procedure was repeated for two more consecutive weeks to measure MO_2_ in fish fed all three experimental diets. Background oxygen consumption was recorded overnight (18 h) in blank (no fish) chambers. Oxygen consumption was not corrected for background respiration as it was considered negligible (< 1% of resting fish MO_2_).

The respirometry chambers used in this study were open to the atmosphere (water exposed to air) meaning O_2_ exchange could have occurred at the surface. Therefore, prior to placing fish into respirometry chambers, experiments were conducted to determine the maximum rate of exchange of O_2_ at the water surface in the current study and its influence on observed rates of fish oxygen consumption. Oxygen was purged from each respirometry chamber down to 50% air saturation by bubbling water with N_2_ and left to re-equilibrate back up to 95–100% air saturation. Re-equilibration was recorded using the respirometry software described above. This revealed that at water O_2_ levels typically observed during respirometry measurements with fish (decline in air saturation from 100 to a minimum of 90%), the rate of O_2_ diffusion from air into the water would have been equivalent to at most 1.8% of the O_2_ removal by fish respiration. Also, this worst-case-scenario rate of O_2_ diffusion would only have occurred when the diffusion gradient was largest between the water and the air, i.e. at the end of the 15-min measurement period. Therefore, the rate of O_2_ diffusion prior to this point, would have been slower and somewhere between zero and 1.8% of the fish respiration rate (MO_2_). Given the negligible impact on rates of oxygen consumption, fish oxygen consumption was therefore not corrected for O_2_ diffusion. This is similar to the conclusions of a previous study by McKenzie et al. (2007)^56^ that used open top respirometry to measure oxygen consumption in rainbow trout maintained at 10 °C. Following a similar test on rates of O_2_ diffusion, their study determined that surface exchange would have modified the decline in O_2_ concentration caused by fish respiration by less than 2%, and they also considered this negligible and not requiring any correction.

If the slope of change in O_2_ over time used to calculate MO_2_ had an R^2^ < 96% it was removed from the data set for that fish. The total SDA was measured as the area under the curve of oxygen consumption rate versus time from the time of feeding until MO_2_ values returned to SMR. The energetic cost of digestion for each individual meal ingested was standardised to kilojoules (kJ) using the total magnitude of the SDA and the conversion factor of 1 mg O_2_ = 14 J^3,57–59^. The SDA scope was calculated by dividing the post-prandial peak in MO_2_ by SMR, and the SDA coefficient (energy cost of digestion relative to the energy content of the meal) was calculated by dividing the total SDA (in kJ) by meal energy.

### Growth

To determine whether an acid buffering diet influenced growth efficiency, 18 fish (n = 6 per diet) were isolated into individual 25 L tanks supplied with dechlorinated freshwater and fed daily on a 1% ration of one of three experimental feeds for 21 days. Initial and final body mass were recorded at the beginning and end of the experimental period and total feed consumed was calculated each day. The feed conversion ratio (FCR) and specific growth rate (SGR; % growth per day) of each individual was then calculated as follows:

2 $$FCR=\frac{total\, mass\, of\, feed\, consumed}{(final\, fish\, mass-initial\, fish\, mass)}$$

3 $$SGR=\frac{In\, final\, weight (g) - In\, initial\, weight (g)}{experimental \,days } \times 100$$

### Statistical analyses

All statistical analyses were performed in R version 4.0.3 (R Development Core Team, 2020) in the RStudio environment Version 1.3.1093–1 ‘Apricot Nasturtium’ (RStudio, Inc 2020). All graphics were produced in Prism Version 9.00 for Mac (GraphPad Software, La Jolla California USA). Analyses were conducted following a D'Agostino & Pearson normality test. The R package ‘fishMO2′^52^ (Version 0.43) was used to determine standard metabolic rate (SMR) and the magnitude, duration and peak MO_2_ of the SDA from each individual (where τ = 0.2, λ = 1). MO_2_ values with an R^2^ < 0.96 were removed from further SDA analyses^55^. A repeated-measures one-way analysis of variance (RM- ANOVA), and Tukey’s multiple comparisons test was used to determine differences in the SDA (total magnitude, duration, peak, time to peak, SDA coefficient and SDA scope) and cumulative acid–base fluxes (ammonia (J_Tamm_), titratable alkalinity (J_Talk_) and net acid or base flux) between diets. A standard one-way ANOVA and Tukey multiple comparisons test was performed to assess differences between diets for changes in mean blood pH, HCO_3_^−^, the partial pressure of CO_2_ (pCO_2_), hourly fluxes of J_Talk_ and J_Tamm_, gut pH and HCO_3_^−^ concentration, the feed conversion ratio (FCR) and specific growth rate (SGR). Where applicable comparisons to fasted animals were conducted using a two-sample t-test. As an additional measure, a linear mixed effects model was used to examine the relationship between total acid-buffering consumed (μmol HCl required to titrate the food ingested (per 100 g of fish) to pH 3.5) and the SDA. Similarly, a simple linear regression was performed to assess the relationship between total acid-buffering consumed, gut pH, HCO_3_^−^concentration, cumulative fluxes, FCR and SGR. Model selection was determined using the AIC function, and where suitable diet order, fish mass, tank and/or individual were included in the model as a fixed or random factor. Data are expressed as means ± SE where *n* = number of fish or samples. Significance was accepted at *P* < 0.05.

## Supplementary Information

Supplementary Information 1.

Supplementary Information 2.

## Data Availability

All data generated or analysed during this study are included in this published article (and its Supplementary Information files—available to download).
