# Supplementary material for: Fish feeds supplemented with calcium-based buffering minerals decrease stomach acidity, increase the blood alkaline tide and cost more to digest
Source: Sci Rep. 2022 Nov 2;12:18468. doi: 10.1038/s41598-022-22496-3 (PMC9630376; doi:10.1038/s41598-022-22496-3)
Supplement: Supplementary file 1 — Supplementary Information 1. [file 41598_2022_22496_MOESM1_ESM.pdf]

Supplementary information for: Fish feeds supplemented with calcium-based buffering minerals decrease stomach acidity, increase the blood alkaline tide and cost more to digest.

Harriet R. Goodrich [1*], Alex A. Berry[2], Daniel W. Montgomery[3], William G. Davison[2], Rod W. Wilson[2*]

[1]Institute for Marine and Antarctic Studies, University of Tasmania, Private Bag 49, Hobart, TAS 7001, Australia

[2] Biosciences, University of Exeter, Exeter, Devon EX4 4PS, UK

[3] Department of Zoology, 4200 - 6270 University Blvd, Vancouver, BC Canada

[*]Author for correspondence (e-mail: harriet.goodrich@utas.edu.au and r.w.wilson@exeter.ac.uk)

Table 1: Output from multiple two-sample t-tests comparing differences from fasted animals in blood HCO_3^-, pH and PCO_2 at 24 and 48 h after juvenile rainbow trout (*Oncorhynchus mykiss*) were fed on a 2.5 % ration of a diet supplemented with calcium chloride ($CaCl_2$), calcium phosphate ($Ca_3(PO_4)_2$) or calcium carbonate ($CaCO_3$). Significance was accepted at $P < 0.05$ and is indicated by *.

Fasted comparisons $CaCl_2$

	HCO_3^-			pH			pCO_2		
Time post feed	Df	t	P	Df	t	P	Df	t	P
24 h	9	0.37	0.72	9	-0.52	0.62	9	0.44	0.67
48 h	10	0.38	0.71	10	-1.19	0.26	10	1.09	0.29

Fasted comparisons $CaCO_3$

	HCO_3^-			pH			pCO_2		
Time post feed	Df	t	P	Df	t	P	Df	t	P
24 h	10	4.3	< 0.01 *	10	1.28	0.23	10	0.13	0.9
48 h	9	1.1	0.29	9	0.13	0.89	9	0.26	0.79

Fasted comparisons $Ca_3(PO_4)_2$

	HCO_3^-			pH			pCO_2		
Time post feed	Df	t	P	Df	t	P	Df	t	P
24 h	10	4.3	< 0.01 *	10	1.2	0.25	10	0.2	0.84
48 h	10	0.72	0.48	10	-0.1	0.92	10	0.23	0.82

Table 2: Output from one-way ANOVA and Tukey multiple comparisons tests comparing differences in blood HCO_3^-, pH and PCO_2 at 24 and 48 h after juvenile rainbow trout (*Oncorhynchus mykiss*) were fed on a 2.5 % ration of a diet supplemented with calcium chloride ($CaCl_2$), calcium phosphate ($Ca_3(PO_4)_2$) or calcium carbonate ($CaCO_3$). Significance was accepted at $P < 0.05$ and is indicated by *.

	HCO_3^-		pH		pCO_2	
	24 h	48 h	24 h	48 h	24 h	48 h
One way ANOVA	F(2,14) = 5.9, P = 0.014 *	F(2,14) = 4.0, P = 0.68	F(2,14) = 3.7, P = 0.048 *	F(2,14) = 2.05, P = 0.16	F(2,14) = 0.09, P = 0.9	F(2,14) = 0.64, P = 0.54
$CaCl_2$ - $Ca_3(PO_4)_2$	0.03 *	0.96	0.09	0.29	0.93	0.57
$CaCl_2$ - $CaCO_3$	0.02 *	0.66	0.05	0.18	0.9	0.66
$Ca_3(PO_4)_2$ - $CaCO_3$	0.91	0.81	0.93	0.93	0.99	0.99

Table 3: Output from one-way ANOVA and Tukey multiple comparisons tests comparing differences in hourly fluxes of ammonia (jTamm) and titratable alkalinity (jTalk) over 6 flux periods after juvenile rainbow trout (*Oncorhynchus mykiss*) were fed on a 2.5 % ration of a diet supplemented with either calcium chloride ($CaCl_2$), calcium phosphate ($Ca_3(PO_4)_2$) or calcium carbonate ($CaCO_3$). Significance was accepted at *P* < 0.05 and is indicated by *.

	JTamm umol kg^{-1} h^{-1}						JTalk umol kg^{-1} h^{-1}					
	Flux 1 (-23 to -1 h)	Flux 2 (0 to 6 h)	Flux 3 (7 to 23 h)	Flux 4 (24 to 47 h)	Flux 5 (48 to 71 h)	Flux 6 (72 to 96 h)	Flux 1 (-23 to -1 h)	Flux 2 (0 to 6 h)	Flux 3 (7 to 23 h)	Flux 4 (24 to 47 h)	Flux 5 (48 to 71 h)	Flux 6 (72 to 96 h)
One way ANOVA	F(2,23) = 0.05, P = 0.95	F(2,25) = 3.5, P = 0.047*	F(2,25) =0.14, P = 0.87	F(2,23) = 0.6, P = 0.55	F(2,27) = 0.26, P = 0.78	F(2,22) = 0.015, P = 0.98	F(2,26) = 0.14, P = 0.86	F(2,19) = 2.92, P = 0.08	F(2,20) = 2.7, P = 0.09	F(2,26) = 3.4, P = 0.045 *	F(2,21) = 0.19, P = 0.82	F(2,23) = 0.18, P = 0.83
$CaCl_2$ - $Ca_3(PO_4)_2$	0.96	0.043*	0.85	0.53	0.78	0.99	0.93	0.3	0.59	0.09	0.99	0.83
$CaCl_2$ - $CaCO_3$	0.96	0.7	0.93	0.8	0.99	0.99	0.99	0.7	0.08	0.06	0.84	0.98
$Ca_3(PO_4)_2$ - $CaCO_3$	1	0.18	0.98	0.86	0.85	0.98	0.86	0.07	0.34	0.95	0.88	0.92

Table 4: Order in which each individual juvenile rainbow trout (n = 8 animals each consuming all three diets) received a 2.5% ration of the $CaCl_2$, $Ca_3(PO_4)_2$ or $CaCO_3$ diet treatment for measurement of the SDA.

**Fish ID**	**Chamber**	**Diet 1**	**Diet 2**	**Diet 3**
A	1	$CaCO_3$	$Ca_3(PO_4)_2$	$CaCl_2$
B	2	$CaCl_2$	$CaCO_3$	$Ca_3(PO_4)_2$
C	3	$Ca_3(PO_4)_2$	$CaCO_3$	$CaCl_2$
D	4	$Ca_3(PO_4)_2$	$CaCO_3$	$CaCl_2$
E	5	$Ca_3(PO_4)_2$	$CaCO_3$	$CaCl_2$
F	6	$Ca_3(PO_4)_2$	$CaCl_2$	$CaCO_3$
G	7	$CaCO_3$	$Ca_3(PO_4)_2$	$CaCl_2$
H	8	$CaCO_3$	$CaCl_2$	$Ca_3(PO_4)_2$
